# Unexpected Long-Term Survival in Resected Pancreatic Ductal Adenocarcinoma Harboring KRAS G12R with an Ultra-Low-VAF SMAD4 Mutation: A Case Report and Mini-Review

**DOI:** 10.3390/reports9030285

**Published:** 2026-08-25

**Authors:** Bianca Varzaru, Adina Emilia Croitoru, Razvan Andrei Iacob, Simona Olimpia Dima, Cristian Gheorghe

**Affiliations:** 1 Faculty of Medicine, Carol Davila University of Medicine and Pharmacy, 020021 Bucharest, Romania; bianca.stoica@drd.umfcd.ro (B.V.); adina.croitoru@umfcd.ro (A.E.C.);; 2 Medical Oncology Department, Fundeni Clinical Institute, 022328 Bucharest, Romania; 3 Digestive Diseases and Liver Transplantation Center, Fundeni Clinical Institute, 022328 Bucharest, Romania; 4 Center of Excellence in Translational Medicine, Fundeni Clinical Institute, 022328 Bucharest, Romania

**Keywords:** pancreatic ductal adenocarcinoma, neutrophil-to-lymphocyte ratio, ccfDNA, KRAS/SMAD4, case report

## Abstract

**Background and Clinical Significance**: Pancreatic ductal adenocarcinoma (PDAC) is an aggressive malignancy with a poor prognosis, even in resectable disease. Molecular alterations in the four major driver genes (*KRAS*, *SMAD4*, *CDKN2A*, and *TP53*) have been associated with disease progression and patient outcomes. However, reliable prognostic biomarkers remain limited, particularly in resected PDAC, where recurrence rates remain high; **Case presentation:** We report the case of a 71-year-old woman incidentally diagnosed with PDAC carrying *KRAS* G12R with an ultra-low-variant allele frequency (VAF) *SMAD4* mutation, with low baseline neutrophil-to-lymphocyte ratio (NLR) and circulating cell-free DNA (ccfDNA) levels. The patient underwent distal pancreatectomy with splenectomy and portal vein resection with multiple postoperative complications requiring re-intervention. The patient demonstrated excellent tolerance to the therapy, experiencing no significant adverse events and reaching an overall survival of 53 months. **Conclusions:** This case highlights the potential value of integrating molecular alterations with inflammatory and liquid biopsy biomarkers, including NLR and ccfDNA levels for improving risk stratification in PDAC. A focused review of the literature further supports the need for multimodal prognostic assessment in resected disease.

## 1. Introduction and Clinical Significance

Pancreatic ductal adenocarcinoma (PDAC) is one of the most aggressive cancers, with an annual death rate approaching its incidence. Despite advances in diagnosis and treatment, most cases are detected at advanced stages, when curative surgery is no longer feasible.

In contrast, incidentally detected pancreatic lesions may allow for earlier diagnosis and surgical intervention. Resected early-stage PDAC and high-risk pancreatic lesions in asymptomatic individuals are associated with improved survival, compared with patients with symptomatic disease, largely due to smaller tumor burden and higher resectability at diagnosis [1].

Genetically, more than 90% of PDAC cases harbor a *KRAS* mutation [2], predominantly with codon 12 alterations, with *KRAS* G12D being the most common [3,4]. *KRAS* variants have previously been associated with more aggressive tumors and worse clinical outcomes in the prognosis of resectable disease [4,5,6,7,8]. The *KRAS* G12R mutation marks a unique form of PDAC that acts differently from other *KRAS* mutations and resists standard targeted treatments [9]. Codon 12 mutations in this cancer swap glycine for aspartate, valine, arginine, or rarely cysteine. Their rough frequencies in PDAC are 40–47% for G12D, 32–35% for G12V, 15–17% for G12R, and 1–2% for G12C [10].

Loss of *SMAD4* function is linked to tumor progression and metastatic dissemination, and represents an established unfavorable prognostic factor for OS [11,12]. Several studies demonstrated that the combination of *KRAS* and *SMAD4* mutations was an independent prognostic factor for progression-free survival (PFS) and overall survival (OS), and patients with both *KRAS* and *SMAD4* mutations have significantly shorter PFS and OS than those without this mutational profile [7,8,12,13,14,15].

Importantly, the co-occurrence of *KRAS* and *SMAD4* mutations is defined as an independent predictor of shorter PFS and OS, suggesting the existence of a high-risk molecular subtype of PDAC with limited therapeutic responsiveness to standard chemotherapy [13].

This article reports a rare case of incidentally detected PDAC harboring a *KRAS* G12R/SMAD4 co-mutation that achieved a prolonged period of stable disease and OS and presents a narrative review of the literature, emphasizing available treatments and their clinical significance. Beyond genomic alterations, emerging biomarkers such as circulating cell-free DNA (ccfDNA) and systemic inflammatory markers, including neutrophil-to-lymphocyte ratio (NLR) have shown potential prognostic value in pancreatic cancer, although their clinical utility remains under investigation.

## 2. Case Presentation

**Figure 1:**
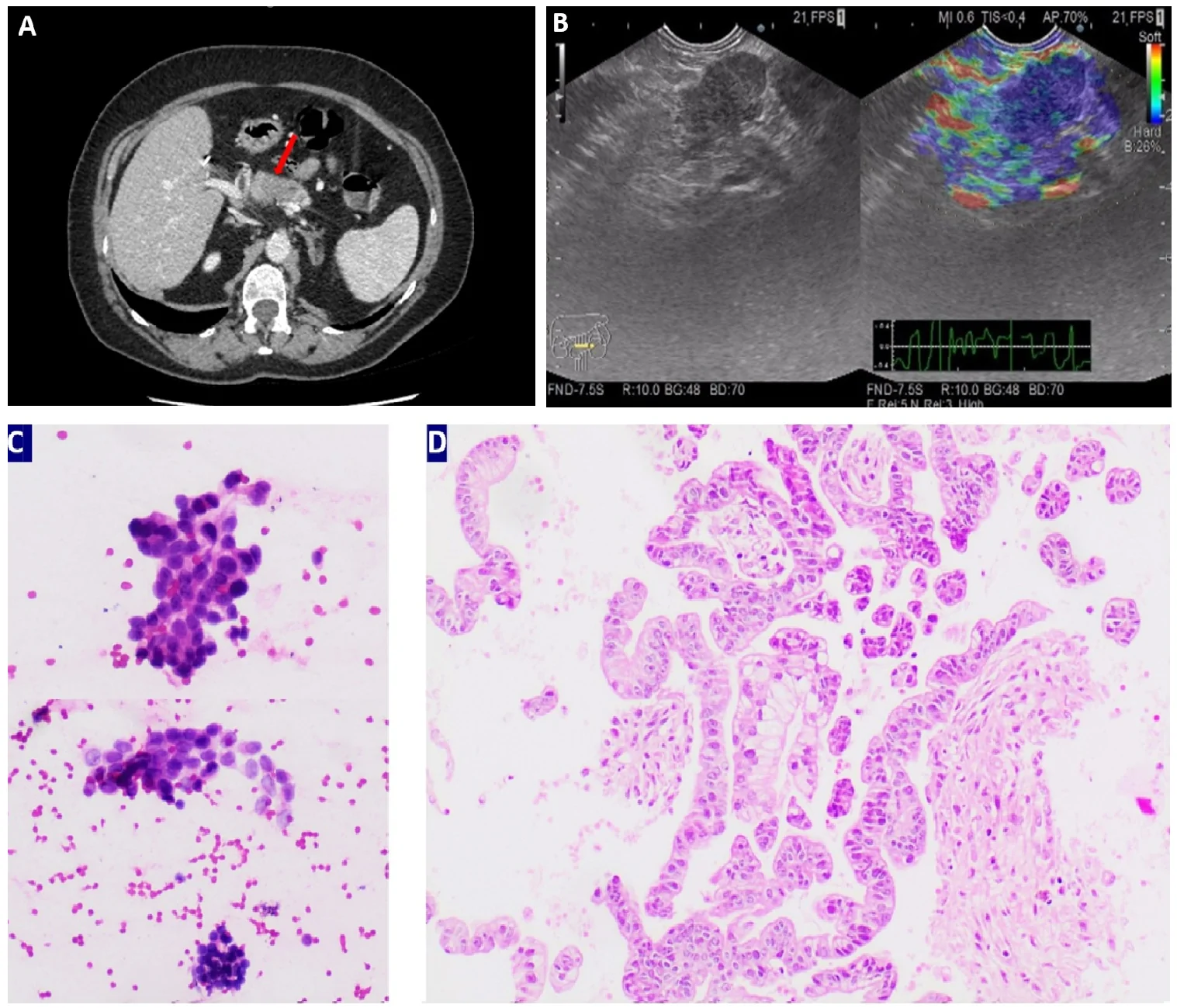
Diagnostic computed tomography (CT) imaging, endoscopic ultrasound (EUS), and histopathological findings: (**A**) a 25 mm pancreatic body tumor in contact with the portal vein confluence without arterial invasion or adenopathy (red arrow). (**B**) EUS and qualitative EUS elastography: a well-defined pancreatic body tumor of 37/17 mm diameter and surrounding large blood vessels; qualitative EUS elastography shows a predominant blue pattern indicating increased stiffness. (**C**,**D**) Hematoxylin–eosin (H&E) staining: atypical cancerous cells that could indicate a low-grade pancreatic adenocarcinoma diagnosis. (**C**) Cytology (H&E stain, 20×): smear with atypical cohesive epithelial cells, tachychromatic nuclei, and reduced pleomorphism. (**D**) Paraffin block (H&E stain, 10×): atypical epithelial flaps with glandular outlines and focal desmoplastic stroma.

A 71-year-old woman with a medical history of hypertension, prior cholecystectomy for gallstone disease, and a family history of rectal adenocarcinoma had a screening computed tomography (CT) colonography in June 2019 that showed a 25 mm mass in the pancreatic body. The tumor was in contact with the portal vein confluence without arterial invasion or adenopathy (Figure 1A).

The patient was admitted to the Gastroenterology Clinic in July 2019. No abnormality was found upon physical examination, and laboratory tests showed normal values of carbohydrate antigen 19-9 (CA19-9), carcinoembryonic antigen (CEA), alpha-fetoprotein (AFP) levels, and total bilirubin. The patient was enrolled in a prospective observational study (PANCNGS: October 2018–December 2021) after providing informed consent. The study was approved by the institutional review board (IRB: 23878/14 June 2018) and included endoscopic ultrasound (EUS)-guided tissue acquisition and fine-needle aspiration (FNA) of the pancreatic mass (Figure 1B) and peripheral blood sampling. EUS-FNA of the pancreatic lesion (Figure 1B) confirmed well-differentiated PDAC (Figure 1C,D).

Genomic DNA (gDNA) and ccfDNA were isolated using the same protocols that we detailed in our prior research [16,17]. The ccfDNA concentration was quantified using a Qubit 3.0 Fluorometer (Life Technologies) and measured in ng/μL, eluted in 25 μL of ultra-pure water. To calculate the ccfDNA concentration per 1 mL of plasma, we multiplied the measured concentration by the elution volume and divided the result by the plasma volume. Based on this calculation, the patient’s ccfDNA concentration was found to be 6.3 ng/mL. The NLR was calculated as the ratio between the peripheral blood neutrophil and lymphocyte counts. The patient’s baseline lab results indicated an absolute neutrophil count of 5030/µL and an absolute lymphocyte count of 2017/µL, yielding an NLR of 2.49. Subsequently, targeted amplicon-based next-generation sequencing (NGS) was carried out using the Illumina NextSeq500 platform. NGS analysis revealed a pathogenic mutation in *KRAS* (G12R), along with alterations in *SMAD4*, *ARID1A*, *TSC2*, *TP53*, *EGFR*, and *ERBB2* (*HER2*), identified in FNA or in both FNA and ccfDNA samples (Table 1).

However, the molecular data could not influence the therapeutic decision, as they were not available at the time of establishing the treatment course.

Following multidisciplinary tumor board evaluation, the disease was classified as resectable PDAC, and surgical resection was recommended.

**Figure 2:**
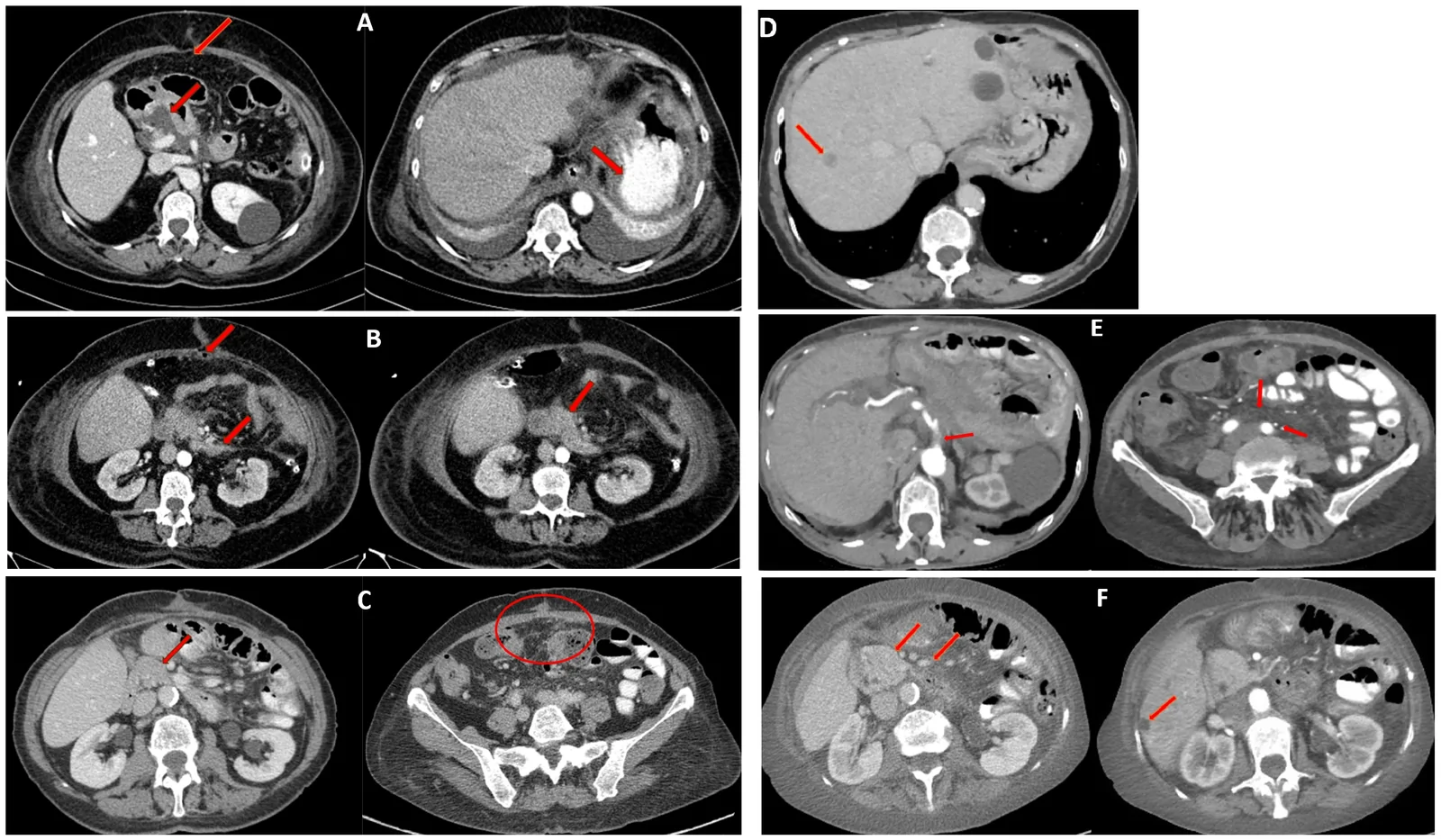
Follow-up CT scans: (**A**) a fistula located on the posterior gastric wall with intraperitoneal extravasation of the oral contrast, intraperitoneal air bubbles, and parafluid collection adjacent to the pancreatectomy site, in the mesogastric and left subphrenic regions; (**B**) no evidence of the previously mentioned small air bubbles, except for one at the level of the laparotomy incision; (**C**) and (**D**) progressive disease: mesenteric and peritoneal infiltration, with adenopathy and peritoneal nodules; (**E**) the tumor involved the superior mesenteric vein, superior mesenteric artery, and celiac trunk; (**F**) dimensional progression of the nodular hepatic lesion and new peritoneal nodules. Red arrows and circles are used to pinpoint and highlight the specific CT anomalies.

On 23 July 2019, the patient underwent a distal pancreatectomy with splenectomy and lateral resection of the portal vein. The corporeo-caudal pancreatectomy specimen (60/30/20 mm) revealed a grayish-white nodular tumor formation (30/20/20 mm) with a histopathological appearance of well-differentiated PDAC, G1 (ductal), and microscopic focal invasion of the anterior and posterior margins (in the peripancreatic fat tissue). Tumor vascular emboli (LVI+) and perineural invasion (PNI+) were visible intratumorally. The architecture of the harvested peritumoral/regional lymph nodes (number 10, dimension 3–5 mm) was intact (pT2N1M0, lymphovascular invasion positive, perineural invasion positive, R1, stage IIB, AJCC 8th edition) [18]. Six days later, the patient developed diffuse abdominal pain and bilious output from the surgical drain. Abdominal CT scan revealed a perforated gastric ulcer with generalized peritonitis (Figure 2A). An emergency surgical re-intervention was performed on 31 July 2019, including segmental gastrectomy, double-layer gastrorrhaphy, peritoneal lavage, and drainage. On postoperative day four following the second procedure, the patient presented with severe abdominal pain, hyperlipasemia (278 U/L, >3× ULN), and increased lipase and amylase in the drain output; these clinical and biological indicators were consistent with the diagnosis of acute pancreatitis of the pancreatic remnant [17].

Following postoperative recovery, adjuvant chemotherapy with gemcitabine monotherapy was started in November 2019 due to the patient’s low performance status (ECOG 2–3). It was continued until April 2020, when the restaged CT scan showed mesenteric and peritoneal infiltration, with adenopathy and peritoneal nodules, indicating progressing disease (Figure 2C,D).

**Figure 3:**
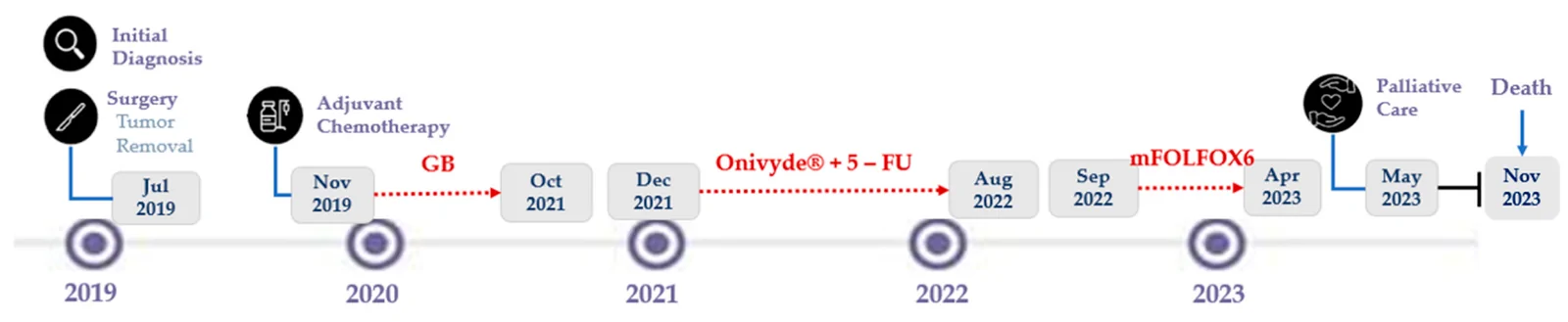
Treatment timeline. GB: gemcitabine-based regimens. Onivyde^®^ + (5-FU): nano-liposomal irinotecan + 5-fluorouracil; mFOLFOX6: modified FOLFOLX-6.

As the ECOG-PS was improved to 0–1, second-line treatment with gemcitabine plus capecitabine was administered from May 2020 to October 2021, achieving stable disease during the initial six cycles. Third-line therapy with nano-liposomal irinotecan (Onivyde, Ipsen Biopharm Ltd, Wrexham, UK^®^) plus 5-fluorouracil (5-FU) was initiated in December 2021 and continued until August 2022, when disease progression involved the superior mesenteric vein, superior mesenteric artery, and celiac trunk, accompanied by rising CA19-9 (114 U/L) (Figure 2E). In September 2022, fourth-line therapy with mFOLFOX6 was started following a multidisciplinary tumor board discussion. The treatment was well-tolerated without notable side effects until April 2023. At that point, the patient’s condition worsened (ECOG-PS 2–3), developing grade I peripheral neuropathy, anemia, and neutropenia, accompanied by a marked increase in CA19-9 (555 U/L). Imaging revealed progression of liver metastasis and new peritoneal nodules (Figure 2F). The patient’s condition progressively declined, and she died in November 2023, after a 53-month OS. The patient’s treatment timeline is illustrated in Figure 3.

## 3. Discussion

A comprehensive literature search was conducted in MEDLINE (via PubMed) and the Scopus database to identify relevant studies investigating the prognostic value of ccfDNA, NLR, and *KRAS/SMAD4* co-mutations in PDAC. The search strategy utilized a combination of Medical Subject Headings (MeSH) terms and text keywords, structured around three core concepts: (1) “Pancreatic Ductal Adenocarcinoma” or “Pancreatic Cancer”; (2) biomarkers and genetic alterations, including “cell-free DNA”, “cfDNA”, “ccfDNA”, “neutrophil-to-lymphocyte ratio”, “NLR”, “KRAS” (G12C, G12D, G12R, G12V), and “SMAD4”; and (3) clinical outcomes, including “prognosis”, “overall survival”, “progression-free survival”, and “chemotherapy responsiveness”. Boolean operators (AND, OR) were applied to combine these fields within a single search string, and title/abstract tags ([Title/Abstract]) were used to maximize sensitivity.

### 3.1. Prognostic Value of ccfDNA Concentration and NLR in PDAC

In a previously published prospective cohort study including 82 patients with PDAC, we demonstrated that higher values of NLR (≥3.31) were linked with worse OS (4 vs. 10 months; log-rank *p* = 0.011), and an elevated ccfDNA concentration (≥25.79 ng/mL) was strongly associated with shorter OS (4 vs. 8 months; log rank *p* = 0.009). According to the results of the multivariable Cox regression analysis, the baseline concentration of ccfDNA was an independent prognostic factor for OS (HR 0.45, 95% CI 0.21–0.97, *p* = 0.041). Furthermore, when ccfDNA levels and NLR were combined, patients with both negative biomarkers (ccfDNA concentration < 25.79 ng/mL and NLR < 3.31) showed significantly longer OS than patients with one or both positive biomarkers [16].

In various studies, multivariate analysis demonstrated that patients with higher NLR (cut-off values ranged from 2 to 5) had a worse prognosis than those with lower NLR [19,20,21,22]. Additionally, a recent analysis involving 61 patients with advanced PDAC demonstrated that ccfDNA concentrations greater than 26.46 ng/mL act as a negative prognostic indicator, correlating with worse overall survival and shorter progression-free survival [23].

Growing evidence indicates that cancer-induced systemic inflammation accelerates tumor progression by driving cell proliferation, metastasis, and angiogenesis [24,25]. The inflammatory response is crucial at every stage of tumor development, from initiation and promotion to malignant conversion, invasion, and metastasis [25,26]. Consequently, several immune- and inflammation-based prognostic tools—such as lymphocyte count, platelet–lymphocyte ratio (PLR), and NLR—are used to assess inflammatory responses linked to poor survival and high recurrence rates across various malignancies, including PDAC [27,28].

Consistent with previous studies, the patient’s ccfDNA levels and NLR suggest a positive biological profile.

### 3.2. KRAS/SMAD4 Co-Mutation’s Impact on Chemotherapy Responsiveness and Survival

About 90% of invasive PDACs harbor *KRAS* mutations, which appear early in the disease course. Although *KRAS* mutations occur too frequently to be considered appropriate prognostic factors, previous reports mention that the *KRAS* mutant subtype G12V or G12D is associated with poor prognosis [29]. Several studies reported that the presence of *KRAS* G12D in the primary tumor was a negative prognostic factor, including within the subgroup that received chemotherapy [30,31,32,33]. The *KRAS* G12R mutation occurs in 12% to 21% of PDAC cases. It involves a glycine-to-arginine swap at codon 12. Compared to G12D and G12V mutations, this variant is associated with reduced MAPK signaling, a less aggressive tumor phenotype, and better patient survival in comparison to other KRAS mutations [10].

The authors of a study involving 1360 consecutive patients who had pancreatic resection for PDAC discovered that *KRAS* G12D mutations had a greater rate of lymph node positivity (in comparison to *KRAS* G12R) and were similarly enriched among early- and late-stage tumors. Additionally, they observed that patients with *KRAS* G12R-mutant PDAC who underwent resection were more likely to have received neoadjuvant chemotherapy (32.7% vs. 17.9% for KRAS G12D, *p* = 0.094) and that patients who underwent upfront resection were less likely to have *KRAS* G12R alterations than those who received neoadjuvant chemotherapy (20.2% vs. 12%; *p* = 0.094) [34]. A greater prevalence of *KRAS* G12D mutations was found in patients with metastatic disease (stage IV) than in patients with localized disease (stage I–III) [35].

When comparing clinical features between *KRAS* mutation subtypes, a greater percentage of patients with G12D, G12V, or G12R mutations showed elevated CA 19-9 levels (77.8%, 85.4%, and 85.2%, respectively); tumors in the wild-type group were more commonly found in the pancreatic head, whereas tumors in the G12R group were more frequently found in the pancreatic body or tail; there was no significant difference in the NLR between the subgroups (*p* = 0.201) [36].

Among the four driver genes, *SMAD4* mutations were found to be a predictor of relapse-free survival (RFS). They were associated with an increased risk of metastasis and a poorer outcome [37]. The negative clinicopathological relevance of *SMAD4* deletion in PDACs was validated by a review and meta-analysis of the literature data [38]. In a retrospective study, *SMAD4*-mutated PDAC was significantly more aggressive than the non-mutated type, being linked to peripheral nerve infiltration (*p* = 0.039), pathological fatty infiltration (*p* = 0.016), a lower recurrence-free survival (*p* = 0.045), and elevated preoperative serum CA19-9 levels (>100 U/mL) [39].

Recent research establishes the loss of the *SMAD4* gene as the most significant predictor of PDAC recurrence and metastasis [40]. The impact of *SMAD4* loss on chemotherapy responsiveness may vary depending on certain treatment regimens and disease stages, even though it is linked to a worse prognosis and shorter survival [11,41,42].

Additionally, it was discovered that the combination of *SMAD4* and *KRAS* mutations was an independent predictor of OS and RFS [13].

Nevertheless, *SMAD4* deletion significantly accelerated the growth of pancreatic tumors when paired with the carcinogenic KRAS mutation, resulting in a marked decrease in survival [15,35,43]. *SMAD4* mutations predict poor response to chemotherapy and are associated with early metastatic spread; they are currently not targetable, but may serve as a biomarker for aggressive disease needing intensified monitoring or alternative therapeutic strategies [11,44]. At the moment of the NGS, the variant read frequency of *SMAD4* mutation was extremely low (0.12%) in comparison to *KRAS* G12R (15.8%), but taking into account the aggressive phenotype that these mutations generate, it could have been highly significant during patient follow-up.

The co-occurrence of *KRAS* and *SMAD4* mutations defines a high-risk molecular subtype, commonly associated with poor prognosis, enhanced metastatic potential, and limited responsiveness to standard chemotherapy [7,8,12,13,14,15]. Yokose et al. demonstrated that the combination of *KRAS* and *SMAD4* mutations is an independent poor prognostic factor for recurrence and survival in resected PDAC [13]. Due to the low VAF of the *SMAD4* variant at the time of sequencing, it is reasonable to assess that the *KRAS* variant was the main prognostic driver in our case.

Preclinical research confirms that specific *SMAD4* and *KRAS* mutations directly shape the surrounding tumor microenvironment. For instance, pancreatic tumors with *KRAS* G12D mutations and *SMAD4* deletion uniquely develop a fibro-inflammatory-rich stroma with increased malignant JAK/STAT cell signaling and improved therapeutic response to JAK/STAT inhibition. Unlike the G12D type, G12V tumors with lost *SMAD4* do not rely on JAK/STAT signaling. This means specific gene mutations change how cancer and surrounding cells behave, which alters how well treatments work [45]. These findings highlight a promising shift toward precision, genotype-directed therapeutics.

We summarized these key genetic drivers in Table 2 to provide a clear overview of how individual and co-occurring mutations dictate survival impact. Table 3 summarizes data from three clinical cohorts evaluating PDAC patients with *KRAS* and *SMAD4* co-mutations, detailing their patient volumes, disease characteristics, treatment regimens, survival outcomes, and key clinical insights.

### 3.3. Impact of KRAS Mutation Subtypes and Co-Occurring Mutations on Treatment Response and Prognostic

*TP53* mutation, frequently observed in PDAC, adds to the aggressive biology, reflecting a high degree of genomic instability and resistance to apoptosis [12,46,47]. Alterations in *ARID1A* and *TSC2* further suggest epigenetic dysregulation and mTOR pathway activation, respectively, both of which are linked to tumor progression and treatment resistance [48,49,50,51].

Although *KRAS* mutations have long been considered “undruggable”, the recent development of G12D-specific inhibitors (e.g., MRTX1133) opens the door for targeted therapy in clinical trials [44,52,53]. Newer multi-RAS or tricomplex inhibitors (such as RMC-6236) are under active clinical evaluation to bypass the structural binding limitations unique to the G12R mutation [10].

*ARID1A* mutations are associated with increased tumor immunogenicity, especially when accompanied by high tumor mutational burden or microsatellite instability [49]. The high frequency of occurrence of these mutations in our case may suggest sensitivity to immune checkpoint inhibitors (e.g., anti-PD-1/PD-L1), particularly in the context of a broader immunogenic signature.

*ERBB2* (*HER2*) mutation cases are suitable for HER2-targeted therapy. If confirmed by IHC or FISH as HER2 overexpression or amplification, this may justify the use of HER2-targeted agents (e.g., trastuzumab, pertuzumab, or trastuzumab deruxtecan) [54,55,56,57].

*EGFR* mutations are of limited significance for PDAC. They require a specific mutational context (e.g., L858R or exon 19 deletion) to be actionable. Historically, EGFR inhibitors (e.g., erlotinib) showed modest benefits in PDAC [58,59]; re-evaluation may be warranted if a sensitizing *EGFR* mutation is present.

Most variants detected in this case (except *KRAS* G12R) were identified at low variant allele frequency (VAF), due to the ultra-deep sequencing strategy used, which is mandatory for sequencing ccfDNA. Due to their low abundance, they have to be interpreted cautiously; however, such mutations may hold significant prognostic and therapeutic relevance, particularly when monitored over time. Thus, these low-frequency variants should not be dismissed, but rather integrated into a longitudinal molecular surveillance strategy using the highly sensitive ddPCR technique, which would enable early detection of clinically relevant emerging subclones. The fact that we were not able to perform this in our study is a limitation.

After decades of failed attempts, several small-molecule *KRAS* inhibitors are currently in development. These include both mutant-specific agents (such as KRAS G12C) and “pan-RAS” inhibitors that target all RAS family members, including wild-type proteins. Although toxicity was a historical concern, recent trials with newer RAS inhibitors demonstrate improved safety profiles and encouraging clinical efficacy, renewing optimism for combination therapies in lung and pancreatic cancers [60].

Postoperative complications following pancreatic surgery are often associated with treatment delays and the omission of adjuvant chemotherapy [61]. In this case, the patient’s PFS (Table 4) under first-line gemcitabine monotherapy was 5 months; under second-line gemcitabine-based regimens, the PFS was 18 months, which was similar to that of the ESPAC4 study [62,63]. Onivyde^®^ with 5-FU third-line therapy produced a PFS of 9 months, above the median PFS of 6.1 months found in the NAPOLI-1 study [64]. Tumor marker levels were also significantly lowered by this third treatment. Additionally, despite their postoperative course, the patient tolerated the treatment well.

The incidental diagnosis of an early-stage (resectable) PDAC with *KRAS* G12R and ultra-low-VAF *SMAD4* alteration, which experienced multiple postoperative complications and a prolonged period of stable disease under adjuvant chemotherapy, both on the first and second lines of chemotherapy, exemplifies this case’s uniqueness. However, early diagnosis and surgery remain the cornerstone of increased OS and PFS, even in the presence of negative molecular markers.

## 4. Conclusions

This case highlights prolonged survival in resected PDAC harboring *KRAS* G12R with an ultra-low-VAF *SMAD4* mutation, a molecular profile that could indicate a poor prognosis. Low baseline NLR and ccfDNA levels may have contributed to a more favorable clinical course. This single case highlights the feasibility of concurrently tracking molecular alterations alongside inflammatory and liquid biopsy biomarkers, such as NLR and ccfDNA levels. While these preliminary observations warrant validation in larger cohorts, they suggest a practical framework for exploring multi-modal risk stratification in PDAC. Integrating molecular and circulating biomarkers may improve prognostic stratification in resected PDAC.

## Figures and Tables

**Table 1:** Molecular profile of the tumor and ccfDNA in the reported PDAC case.

Gene	Alteration	Detection Context	Potential Clinical Relevance
*KRAS*	G12R mutation p. (Gly12Arg) COSM518	High-confidence pathogenic driver, detected in FNA gDNA	Key oncogenic driver associated with aggressive PDAC biology and poor prognosis
*SMAD4*	p. (Ala39Val) COSM6475969	Low-frequency variant detected in both FNA and ccfDNA, but not in corresponding leukocyte gDNA	Tumor suppressor loss is linked to metastatic potential and reduced survival
*TP53*	Missense variant Stop-gained Frameshift variant	Detected in tumor tissue and ccfDNA	Genomic instability contributes to tumor progression
*ARID1A*	Inframe deletion Frameshift variant	Subclonal/low-frequency variant	Epigenetic dysregulation: potential role in tumor aggressiveness
*TSC2*	Frameshift variant Missense variant Stop-gained	Subclonal variant/low-frequency variant	Possible activation of the mTOR pathway
*EGFR*	Missense variant	Subclonal/low-frequency variant	Limited actionable relevance in PDAC unless the sensitizing mutation is confirmed
*ERBB2* (HER2)	Missense variant Stop-gained Frameshift variant	Subclonal/low-frequency variant	Potential therapeutic relevance if confirmed amplification/overexpression

Abbreviations: ccfDNA: circulating cell-free DNA, PDAC: pancreatic ductal adenocarcinoma; VAF: variant allele frequency.Variants detected at low VAF likely reflect tumor heterogeneity and ultra-deep sequencing sensitivity. Clinical significance of these alterations should be interpreted in the context of longitudinal molecular monitoring.

**Table 2:** Prognostic Significance of *KRAS* and *SMAD4* Mutations.

Genetic Alteration	Type of Alteration/Variant	Prognostic Significance/Impact on Survival
*KRAS* Mutation	Pathogenic mutation (predominantly *KRAS* G12D)	Associated with aggressive tumors, poor clinical outcomes, and reduced overall survival.
*SMAD4* Mutation	Loss of function/Deletion/Loss	Linked to tumor progression and metastatic dissemination, and serves as an unfavorable prognostic factor.
*KRAS* & *SMAD4* Co-Mutation	Combined *KRAS* and *SMAD4* loss/mutation	Acts as an independent poor prognostic factor for progression-free and overall survival, presenting significantly shorter survival times.

**Table 3:** Summary of Clinical Cohorts Evaluating *KRAS*/*SMAD4* Co-Mutations in PDAC.

Study	Patients (*n*) with *KRAS/SMAD4* Co-Mutation	Disease Characteristics	Treatment	PFS/RFS/TTNT	OS	Key Clinical Insights & Co-Drivers
Yokose et al., 2020 [13]	*n* = 15 (30% of a 50-patient sequenced cohort)	Untreated, pathologically confirmed resectable PDAC.	Upfront radical surgical resection followed by systemic adjuvant chemotherapy.	12.3 months (Median RFS)	22.3 months	Combined *KRAS* and *SMAD4* mutations function as a highly aggressive independent prognostic factor for early recurrence and reduced survival.
Yousef A. et al., 2024 [35]	*n*** = 65** (11.23% of the *n* = 578 *KRAS*) multi-institutional validation cohort	Mixed staging; 42% documented baseline metastatic disease	Standard first-line cytotoxic combinations (FOLFIRINOX vs. Gemcitabine/Nab-Paclitaxel).	~3.5 to 5.1 months (Median across cytotoxic lines)	14.0 months (Median OS for the primary *KRAS* group)	Showed extreme resistance to traditional systemic lines; validation highlighted *KRAS* (92%) and *SMAD4* (24%) as core drivers.
Norton et al., 2024 [9]	*n* = 89 (*KRAS* G12R/*SMAD4*)	Metastatic PDAC	Standard first-line cytotoxic combinations (FOLFIRINOX vs. Gemcitabine/Nab-Paclitaxel).	~3.8 to 6.2 months (Median TTNT across cytotoxic lines)	~8 to 13.2 months (Median OS across cytotoxic lines)	*KRAS* G12R was associated with more favorable clinical outcomes than other *KRAS* mutations and had the highest incidence of comutations with *SMAD4*.

Abbreviations: PDAC: pancreatic ductal adenocarcinoma, PFS: progression-free survival, RFS: relapse-free survival, TTNT: time to next treatment, OS: overall survival.

**Table 4:** Systemic therapy summary.

Line	Regimen	Duration	Response	PFS (Months)
**1st Line**	Gemcitabine monotherapy	November 2019–April 2020	**Disease Progression**; ECOG-PS improvement (from 2–3 to 0–1)	5
**2nd Line**	Gemcitabine + Capecitabine	May 2020–October 2021	**Stable Disease** (Initial 6 cycles); ECOG-PS 0–1	18
**3rd Line**	Onivyde^®^ + 5-FU	December 2021–August 2022	**Disease Progression** involving celiac trunk, SMV, and SMA; ECOG-PS 0–1	9
**4th Line**	mFOLFOX6	September 2022–April 2023	**Disease Progression**; induced neuropathy, anemia, and neutropenia; ECOG-PS 2–3	7

Abbreviations: SMV: superior mesenteric vein, SMA: superior mesenteric artery.

## Data Availability

The original contributions presented in this study are included in the article. Further inquiries can be directed to the corresponding author.

## Abbreviations

The following abbreviations are used in this manuscript:

PDAC	Pancreatic Ductal Adenocarcinoma
NLR	Neutrophil-to-lymphocyte ratio
ccfDNA	Circulating cell-free DNA
OS	Overall Survival
PFS	Progression-Free Survival
CT	Computed Tomography
CA19-9	Carbohydrate Antigen 19-9
CEA	Carcinoembryonic Antigen
AFP	Alpha-Fetoprotein
EUS-FNA	Endoscopic Ultrasound-Guided Tissue Acquisition and Fine Needle Aspiration
gDNA	Genomic DNA
NGS	Next Generation Sequencing
5-FU	5-fluorouracil
RFS	Relapse-free survival
VAFs	Variant allele frequencies
TTNT	Time to next treatment
